# Percutaneous bone adhesive application for Jones fracture “at-risk” of nonunion or delayed union: a hypothesis

**DOI:** 10.1186/s13037-022-00348-3

**Published:** 2022-12-02

**Authors:** Niaz Ahankoob, Vincent P. Stahel

**Affiliations:** 1 Rocky Vista University, College of Osteopathic Medicine, 8401 S. Chambers Rd, CO 80134 Parker, USA; 2 University of Colorado Boulder, CO 80309 Boulder, USA

**Keywords:** Bone adhesive, Closed fracture, Fracture displacement, Patient safety, Nonunion

## Abstract

**Background:**

Bone adhesives have been on the forefront of orthopedic surgery research for decades due to the potential benefit they may have in fracture management. Current publications and research being conducted on bone adhesive could be applied to our current hypothesis for the benefit of a novel minimally invasive treatment option for a select cohort of fractures, Jones fractures. The select fracture’s gold standard of treatment would be nonoperative, but with risk of complications including nonunion and delayed union.

**Presentation of hypothesis:**

We hypothesize that percutaneous application of bone adhesive will provide an additional treatment option for fracture patterns that do not require operative fixation, but would benefit from additional stability. The primary outcome measures would be (1) duration of time required for bony consolidation (defined as 3 of 4 bridging cortices) and (2) duration of absenteeism (inability to work), and pain levels within the first week after the procedure. Secondary outcome measures would be the incidence of nonunion or delayed union. We hypothesize that the select bone adhesive would accelerate bony consolidation, decrease absenteeism, decrease pain levels within the first week after procedure, and decrease the incidence of delayed union and/or nonunion.

**Testing of hypothesis:**

We propose a prospective multicenter, randomized, and open label trial clinical trial to test the bone adhesive via percutaneous injection into acute non-displaced or minimally displaced Jones fractures.

**Implications of hypothesis:**

Bone adhesives are a new frontier in treatment of fractures, currently in laboratory and animal testing phases. The appropriate bone adhesive formula has not been approved for clinical trial use, but the implications of the bone adhesive may go beyond decreased complications and ease of stabilizing a select cohort of closed fractures. With the injectable compound illustrated (Fig. 1), the adhesive could be applied percutaneously in hopes of achieving improved outcomes compared to non-operative treatment. The overall goal of the clinical trial is to provide patients a safe treatment option for improved bone union rates of nonoperative fractures compared to the current gold standard management of the same fracture with earlier pain control, early bony consolidation and lower risk of delayed union/nonunion. The ideal patient population for use of a percutaneous bone adhesive in future studies would be for those with multiple medical comorbidities for whom surgical risks outweigh the benefits, in addition to patients at high risk for nonunion based on fracture pattern or systemic biology.

## Background

The concept of a “bone glue” that could replace traditional mechanisms of osteosynthesis, including plates, pins, and screws, has been on the forefront of orthopedic surgery research for several decades [1]. Bone adhesives are a highly attractive and unique method of fixing certain fracture types with potentially mitigated surgical risks [1]. Despite significant research and progress in the field, a compound that adheres to all of the requirements proposed for a bone adhesive to be practical in the clinical setting remains lacking [2].

The comprehensive and strict guidelines regarding the requirements of a bone adhesive (Table 1) can be summarized as biocompatibility, degradability, and sufficient bond strength [2]. The two main groups of adhesives currently being tested in animal studies are divided into synthetic based and biologically-derived, each with their respective current shortcomings [2]. The range of adhesive agents currently available for surgical application including calcium phosphate cements, cyanoacrylates, polyester cements, polymethylmethacrylate (PMMA) cements, and fibrin based adhesives [3]. Each adhesive agent has favorable characteristics, as well as individualized limitations [1]. The adhesive strength of certain agents is promising for orthopedic application in fracture stabilization. For example, polysaccharides have demonstrated excellent adhesion strengths of 40–45 MPa, while other adhesives such as fibrin have displayed adhesion strengths of 0.005–0.17 MPa and appear to be significantly less favorable in this application [3]. The shear strength of a cyanoacrylate based adhesive demonstrated increased strength (1–2 MPa) compared to the shear strength of a plate and screw system (0.49 MPa), implying potential advantages over more typical mechanisms of osteosynthesis [3]. However, many of these adhesives are lacking in regards to other properties, such as adhesion in wet environments, which is why forthcoming research has shifted focus to biomimetic based adhesives [3]. These biomimetic agents have shown to have high levels of bond strength in addition to strong adhesive properties in wet environments [3]. Another type of adhesive, polyurethane based adhesives, have displayed several promising characteristics for application in the field of orthopedic surgery, including ultraviolet light activation, biocompatibility, and a high degree of adhesion strength but were limited by toxicity [3].

**Table 1:** Properties for a bone adhesive modified from “Bone adhesives for trauma surgery: A review of challenges and developments,” by Farrar, 2012, International Journal of Adhesion and Adhesives, 33:89–97 [2], with the additional proposed requirements for successful percutaneous injection

Properties for Bone Adhesive	Additional Requirements
• High level of adhesion to bone, often in the presence of contaminants such as fats, proteins, etc. • Bonds to wet surfaces/bond strength stable in wet environment • Mechanical stability under tension, compression, shear • Easy/quick to prepare and apply in operating room conditions • Adequate working time for the surgeon to apply and form bone • Rapid setting time (typically 1–10 min) • Low exotherm on setting – no thermal necrosis • Non-toxic and biocompatible (including any leachables, degradation products, etc.) • Allows healing of the fracture • Sterilisable • Adequate shelf-life • Cost effective to use • Commercially viable to manufacture • Adhesion to surgical allows (e.g. stainless steel, Ti-6Al-4 V, Co-Cr-Mo, etc.) • Biodegradable in a controlled manner and timescale • No special storage conditions (stable at room temperature) • Ability to deliver drugs/bioactive agents e.g. stimulate bone healing, prevent infection, etc.	• Addition of fluoroscopic-compatible agents • Not overly viscous to block or slow application through the needle • Adequate working time for the surgeon to inject the adhesive • Compatible with use in conjunction with local anesthetic. • Maintains sterility through the process of injection. • Osteoinductive and osteoconductive properties

Though the perfect bone adhesive is still not approved for use in human subjects, there is great merit in exploring the potential benefits. Advantages of bone adhesives over traditional mechanisms of osteosynthesis include eliminating subsequent implant removal operations, minimizing general anesthesia, eliminating implant-related infection rates and potentially decreasing direct and indirect costs [1, 2]. As of now, it is still premature to hypothesize that a bone adhesive has the ability to replace all other surgical fixation options or devices for fracture management. The purpose of this hypothesis paper is to describe a novel intervention that can improve outcomes and decrease possible complications in select patient population for one fracture pattern.

We hypothesize that minimally invasive percutaneous injection of a bone adhesive may potentially provide a safe and effective alternative treatment option that augments bone healing in non-displaced Jones fracture in a healthy patient population. These non-displaced fractures would otherwise be treated nonoperatively but at-risk for nonunion or delayed union.

## Presentation of the hypothesis

**Fig. 1:**
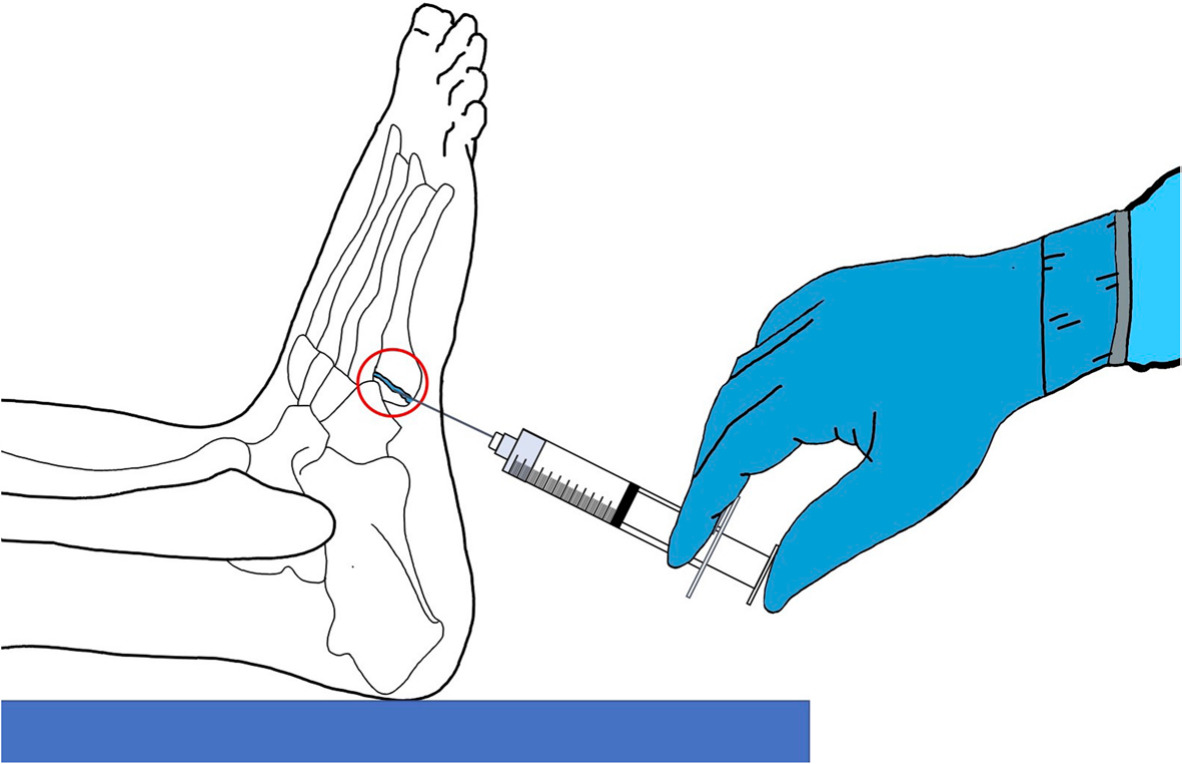
Simplified illustration of a potential indication for percutaneous bone adhesive injection into the fracture site of a non-displaced Jones fracture as to reduce the risk of a delayed union or nonunion

We hypothesize that percutaneous injection of bone adhesive through a needle and syringe (Fig. 1) will lead to comparable outcomes of fracture healing in patients who suffer from closed Jones fractures generally treated nonoperatively, but have an increased risk of nonunion or delayed union. The outcomes to be analyzed include (1) duration of time required for bony consolidation (defined as 3 of 4 bridging cortices) and (2) duration of absenteeism (inability to work), and pain levels within the first week after the procedure. Secondary outcome measures would be the incidence of nonunion or delayed union.

## Testing of the hypothesis

To test this hypothesis, the ideal bone adhesive would first need to be approved for clinical trials in human subjects. A prospective multicenter, randomized, and open label trial on a cohort of at least 30 patients with qualifying Jones fractures is proposed as the ideal clinical trial design (Table 2). The subject would be randomized into either the control group, treated with the gold standard management of that fracture, or the experimental group treated with percutaneous injection of bone adhesive, closed reduction, and adjunctive immobilization as indicated (cast or stiff soled shoe).

**Table 2:** Overview of Jones fracture for treatment with percutaneous bone adhesive injection

**Fracture Type**	Non-displaced 5th metatarsal base (Zone 2) fracture
**Common Injury Mechanism**	Adduction force to the foot with a lifted heel
**Indication for non-operative management**	Non-displaced Zone 2 (Jones) in non-athletes
**Non-operative treatment protocol**	Cast immobilization
**Non-operative recovery period**	6–8 weeks
**Rate of non-operative complications**	15–44% nonunion rate [4, 5]

### Inclusion criteria

Inclusion criteria includes: 18 years and older; closed fracture; Jones fracture (5th metatarsal base fracture in zone 2).

### Exclusion criteria

Exclusion criteria includes: open fracture; immunocompromised patients, HgA1C > 9%; wounds or abrasions in area of percutaneous injection; dermatitis or psoriatic manifestations in area of injection; known allergy to components of bone adhesive.

### Primary outcome parameters

The duration of time required for bony consolidation (defined as 3 of 4 bridging cortices), duration of absenteeism (inability to work), and pain levels within the first week after the procedure.

### Secondary outcome parameters

Secondary outcome measures would be the incidence of nonunion or delayed union, range of motion, pain levels, return to daily activity. Patients will be followed clinically for up to 1 year after date of injury and assessed for radiographic outcomes (maintenance of reduction, callus formation, fusion), functional outcomes (Visual Analog Scale, Patient-Reported Outcomes Measurement Information System), and rate of mortality/complications within one year of trauma, including infections, thromboembolic events, and acute respiratory distress syndrome.

The hypothesis may be proven by demonstrating an improved rate of bone union, lower incidence of nonunion or delayed union, and improved long term outcomes in the experimental group, compared to the control group.

### Proposed requirements for ideal injectable compound

The proposed requirements for a bone adhesive to be practical in the clinical setting was first listed by Farrar [2]. We propose additional requirements in order for the bone adhesive to be successfully applied percutaneously (Table 1). One of the most important requirements, which may pose a challenge, is the addition of an agent into the bone adhesive which would present as radiopaque under fluoroscopic and plan radiograph imaging when initially injected but become radiolucent when cured. This is so that the adhesive could be applied as precisely as possible while allowing the surgeon to follow the bone healing progress appropriately throughout the trial.

### Proposed application method

First, the patient must be properly draped and the extremity must be prepared in a sterile fashion. Then, the surgeon would use fluoroscopic imaging to localize the fracture and to determine the best location for needle insertion and trajectory. Next, the surgeon would insert the needle into the fracture site, taking caution to avoid major neurovascular structures, with the use of fluoroscopic imaging. Once the needle is within the fracture site, the adhesive would be carefully injected by the milliliter while simultaneously taking fluoroscopic images to visualize the amount of the injected adhesive. Lastly, the needle would be withdrawn and the fracture would undergo manipulation for closed reduction. Another image at this time would be beneficial to confirm proper reduction. The surgeon would hold the fracture reduction until the adhesive has cured. A final image would be taken to confirm maintenance of reduction.

## Implications of the hypothesis

As previously mentioned, there is risk of nonunion and delayed union in non-operative management of Jones fractures. The further outcome of these complications includes additional laboratory testing, office visits, hospital stays, surgical intervention, disability, pain and more, which can leave a financial and psychological burden on the patient in addition to increasing risk for jeopardizing patient safety [6]. There are no specific studies looking at financial burden for Jones fracture nonunions, but a few studies have been completed for long bone fractures which demonstrated average $25,000 USD per isolated limb fracture nonunion [7]. These costs do not include the cost related to absenteeism for the patient, such as the inability to work. This novel intervention would provide stability to the fracture, preventing the interfragmentary motion between fracture fragments until a soft callus forms around 2 weeks, thus decreasing early pain and preventing absenteeism. Between operative and non-operative treatment options, there is room for development of a new safe technique to manage closed fractures, such as a minimally invasive percutaneous bone adhesive injection. Though we hypothesize percutaneous injection to be generally safe, it is not without risks such as infection and damage to important neurovascular structures.

The risk of using this injectable compound includes the possibility of introducing infection to the fracture site, which would require further treatment with antibiotics, and irrigation and debridement. It could be assumed that under sterile conditions there is a low risk for infection considering the low risk of infections after insulin injections, vaccination injections and even the invasive epidural injection [8, 9]. In comparison, the risk of a surgical site infection from open surgical fixation is approximately 1.07% in the U.S. in the healthy patient population, and increases with immunocompromised patients. [10, 11]. Though minimal, the risk of infection from a minimally invasive injectable compound may be comparable to the risk of infection from open surgical fixation, and should still be taken into consideration.

Insertion of a needle into the fracture site also has the potential to damage important neurovascular structures along the trajectory. This raises the necessity of having a thorough understanding of anatomy and possibly acquiring ultrasound skills to visualize these structures. In comparison to open reduction and internal fixation, using a needle has the potential to avoid soft tissue damage due to mitigating the risks involved with cauterizing soft tissues, retracting neurovascular structures, and stripping periosteum. With a minimally invasive percutaneous injection it should be possible to decrease soft tissue damage while providing temporary stability in order for the fracture to heal.

There is also continued risk of adhesive toxicities and shortcomings including cytotoxicity, mechanical failure, stress shielding, and late nonunions [3]. In prior studies, adverse events have been noted with the application of bone adhesives including cardiac arrest and hypotensive episodes [12]. Other noted complications include loosening and osteolysis resulting from fragmentation of adhesive [12]. The use of adhesive itself may potentially cause a nonunion or delayed union as it could interfere with the biologic mechanotransduction and/or bony physiology. This is why biodegradability, non-toxicity to surrounding tissue, and quality of biologic ingrowth is an important characteristics for an adhesive [2].

Furthermore, most of the fracture patterns would likely require adjunctive immobilization with nonoperative management, so adjunctive immobilization would pose a risk regardless of the treatment modality.

The purpose of this hypothesis is not to discredit the use of devices, implants or open reduction and internal fixation in fracture stabilization, but rather facilitate conversation regarding investigation of a different treatment option especially for those who are poor surgical candidates. Possible patient candidates include those with greater than or equal to ASA III, poorly controlled diabetes mellitus, morbid obesity and more [13]. Those who are not surgical candidates face the risks associated with nonoperative management of fractures that require operative fixation, which includes malunion or nonunion of fracture, slow rehabilitation, limb deformity, disability, pain, and increased mortality [14, 15]. Percutaneous injection of bone adhesive for treatment of fractures in vulnerable patient populations may be of interest for further investigation in order to improve patient safety.

## Conclusion

A minimally invasive percutaneous bone adhesive injection method and clinical trial to test its efficacy in the clinical setting was introduced. The main focus is on treating fracture patterns, such as the Jones fracture, which are at high risk of nonunion or delayed union with nonoperative treatment. With the percutaneous injection, it could be possible to avoid risks of operative complications, and any additional financial, physical and emotional burden that would come with a nonunion. This hypothesis is a call for the development of new fixation techniques that would better serve patient safety, optimize recovery time, and provide improved clinical outcomes. Once bone adhesives are approved by the Food and Drug Administration, they could be further tested with this injectable design. Thus, this hypothesis study is intended as a basis for increasing discussion for a novel method to manage fractures that would be an addition to the current gold standard treatments currently utilized.

## Data Availability

The data sets used and/or analyzed during the current study are available from the corresponding author on reasonable request.
